# Behavioral responses of skipjack tuna to diluted waters from the Yangtze River in the East China Sea

**DOI:** 10.1371/journal.pone.0356256

**Published:** 2026-10-14

**Authors:** Soya Sakuma, Hiroto Abe, Takayuki Matsumoto, Yuichi Tsuda, Hidetada Kiyofuji

**Affiliations:** 1 Graduate School of Fisheries Sciences, Hokkaido University, Hakodate, Hokkaido, Japan; 2 Faculty of Fisheries Sciences, Hokkaido University, Hakodate, Hokkaido, Japan; 3 Fisheries Resources Institute, Japan Fisheries Research and Education Agency, Yokohama, Kanagawa, Japan; 4 Headquarter, Japan Fisheries Research and Education Agency, Yokohama, Kanagawa, Japan; Hawaii Pacific University, UNITED STATES OF AMERICA

## Abstract

This study investigated changes in the swimming behavior of skipjack tuna (*Katsuwonus pelamis*) in the low-salinity waters of the East China Sea (ECS), influenced by nutrient-rich freshwater from the Yangtze River, to elucidate their response to riverine freshwater. Behavioral data from 18 individuals collected through archival tag release surveys conducted around Japan’s Nansei Islands were analyzed. Five individuals that experienced sea surface salinity (SSS) below 33.5 psu were identified as low-salinity-exposed individuals, and their relationships with marine environmental parameters (e.g., temperature, salinity, chlorophyll, sea surface height, and dissolved oxygen [DO]) were examined at the individual level. Furthermore, energy intake was estimated from body temperature changes, enabling comparison of swimming behavior between low-salinity environments and the open ocean from both physical and physiological perspectives. The five low-salinity-exposed individuals showed different distribution and behavioral patterns between spring (three individuals: Sp1, Sp2, and Sp3) and summer (two individuals: Su1 and Su2). In spring, skipjack tuna were observed in waters with temperatures no less than 18°C. In summer, individuals were observed in environments with DO levels mainly higher than 3.6 mL L^−1^ and SSS of no less than 31.3 psu. Additionally, two individuals (Sp1 and Su2) showed higher estimated energy intake in low-salinity waters compared with the open ocean (Sp1: 139.9 ± 46.0 vs. 92.3 ± 43.8 kcal/day; Su2: 210.5 ± 52.2 vs. 176.6 ± 59.3 kcal/day), while the remaining three individuals showed no significant difference or could not be compared due to very short residence time in low salinity condition. Although these results were obtained from only five individuals, the findings suggest that diluted water in the ECS may affect feeding opportunities and distribution of skipjack tuna through variability in temperature, salinity and DO.

## Introduction

Skipjack tuna (*Katsuwonus pelamis*) is a top predator widely distributed from tropical to temperate regions and supports major multinational fisheries [1–4]. Thus, understanding its ecological characteristics and migratory behavior is crucial for predicting fishing ground formation [5,6], developing resource management strategies [2], and evaluating climate change impacts on fisheries [2,7,8].

Ecological research on skipjack tuna has been conducted worldwide (Fig 1), including studies in the Pacific Ocean [9–15], Atlantic Ocean [16,17], Indian Ocean [17–20], and the global ocean [21]. Marine fish species are poikilotherms, and water temperature affects their metabolic processes and muscle function [22]. Additionally, they are subject to osmotic pressure, which influences their physiological state [23,24]. For skipjack tuna, salinity is therefore considered one of the key environmental factors affecting their physiological condition [25]. Skipjack tuna have high metabolic demands and therefore require environments with high dissolved oxygen (DO) concentrations [26]. Therefore, understanding their habitat environment is essential for understanding skipjack ecology.

**Fig 1:**
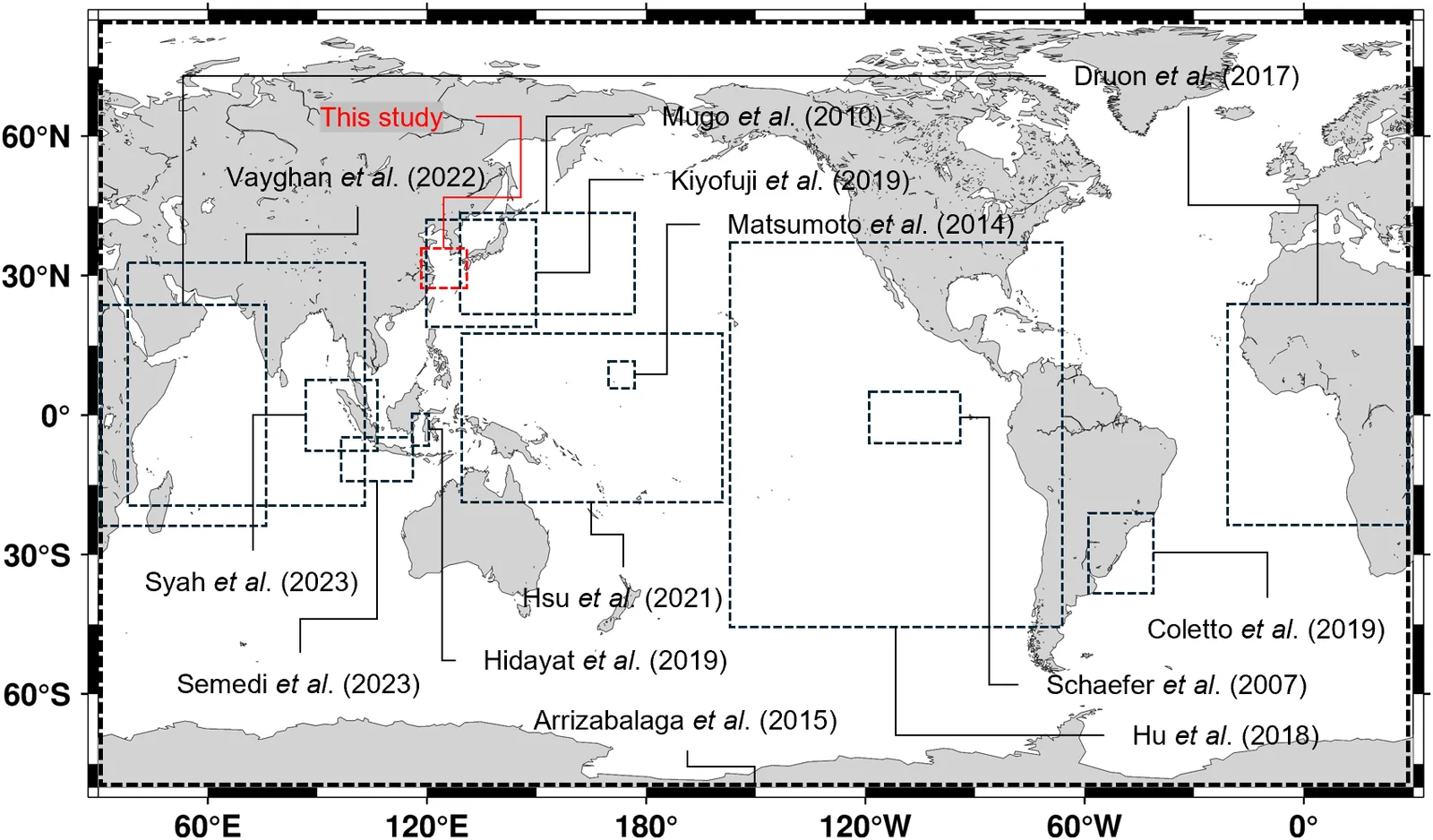
Target areas of previous studies on the distribution and migration of skipjack tuna. Black dotted lines indicate the respective target regions. Red dotted lines indicate the study region.

Fishery-based studies have provided complementary information on environmental preferences in the Pacific Ocean [5,12–14], Atlantic Ocean [16,17], and Indian Ocean [17–20]. For example, studies in the Pacific Ocean indicate that skipjack primarily inhabited waters of 20.5°C–26.0°C [9] in the northwestern region; ≥ 29.6°C with salinity of 34.29–35.28 psu in the western equatorial region [12]; and 20°C–28°C with DO concentrations of 1–4 mL L^−1^ at 150-m depth in the eastern tropical region [14]. In the eastern central Atlantic Ocean and western Indian Ocean, skipjack tuna preferred sea surface temperature (SST) of 21.6°C–30.0°C, sea surface salinity (SSS) of 30.3–36.2 psu, and a minimum DO of 4.4 mL L^−1^ [17], whereas optimal SST of 22°C–24.5°C and salinity of 34.8–35.8 psu were found off Brazil [16]. Global longline data analysis further revealed preferences for surface temperatures of 20°C–28°C with salinity of 36–37 psu [21]. Collectively, skipjack tuna inhabits a wide temperature range of 16°C–32°C and a salinity range of 30.3–37 psu and requires DO levels above ~1–4.4 mL L^−1^. However, most investigations have focused on oceanic regions with relatively limited variability in water quality. In contrast, some coastal regions experience substantial environmental changes driven by river discharge. Therefore, individual behavioral responses to such dynamic conditions remain poorly understood.

The East China Sea (ECS) is located along the path of the Kuroshio Current, which has been identified as a migratory route for skipjack tuna [10]. However, the ECS is among the most environmentally dynamic regions globally, driven by the Yangtze River discharge from the west and the Kuroshio Current from the east [27,28]. During summer, freshwater from the Yangtze River spreads across the ECS, forming a halocline between low-salinity waters in the upper layer and high-salinity waters in the lower layer, resulting in density stratification [29]. This tight stratification, with a density difference of ~3.5 kg m^−3^, inhibits oxygen supply from the ventilated surface water to deeper layers [30]. Along with oxygen consumption through organic matter decomposition by microorganisms and respiration by marine organisms, hypoxic conditions (DO < 2 mL L^−1^) develop beneath the river-diluted water [30–32]. Additionally, the Yangtze River freshwater carries nutrients from agricultural fertilizers washed in from land [33], fueling and supporting massive primary productivity [32,33]. These environmental changes induced by the Yangtze River discharge are expected to substantially influence the ecology and swimming behavior of skipjack tuna in the ECS. However, the responses of skipjack tuna to these environmental changes remain unclear. Although riverine influences have been reported to create favorable feeding environments and positively affect catches in coastal waters off Brazil [16], fishery data reveal distributional patterns but cannot capture individual-level behavioral responses. Therefore, direct tracking approaches are necessary to investigate the individual responses of skipjack tuna to such dynamic environmental conditions.

In recent years, electronic tagging has been widely adopted to investigate the habitats and swimming behavior of many marine organisms, including skipjack tuna [34–38]. Archival tags record not only the swimming positions of tagged fish but also ambient water and body temperature changes. In addition, energy intake can be estimated from changes in body temperature [39,40]. Therefore, release surveys using electronic tags enable the simultaneous consideration of ambient environmental conditions and physiological responses.

Meanwhile, ocean observation methods have diversified, and extensive environmental data have been accumulated, enabling detailed analyses of skipjack tuna habitats. Satellite observations provide key physical data, such as SSS, which indicates regions influenced by river water [41,42], as well as sea surface temperature (SST) and sea surface chlorophyll (SSC). For vertical ocean structures, ~ 4,000 ascending and descending Argo floats are currently deployed globally, collecting profiles of temperature, salinity, and, more recently, DO [43]. DO data are available in the climatological oceanic dataset of the World Ocean Atlas (WOA). Although the WOA does not capture interannual variability, it enables the investigation of seasonal variations in DO.

In this study, we aim to elucidate the relationship between skipjack migratory behavior and the ocean environment influenced by the Yangtze River in the ECS from both physical and physiological perspectives using archival tag data.

## Materials and methods

### Tag data and study area

This study presents a descriptive analysis based on archival tag data from a limited number of individuals (n = 18) obtained through release surveys. Between 2012 and 2016, 628 skipjack tuna were captured by trolling or jigging in the waters around the Nansei Islands, southwestern Japan (Fig 2a). They were measured for fork length (FL), and the 18 recaptured individuals used in this study had a fork of 39.0–49.0 cm at the time of release. They were implanted with archival tags in the peritoneal cavity and subsequently released. 18 individuals were recaptured, and their tags were successfully recovered (recapture rate: 2.9%). Basic data for these individuals, including positional information at release and recapture, dates, and FL, are summarized in Table 1. The archival tags used were LAT2910 tags manufactured by Lotek Wireless Inc. (Canada), measuring 26 mm in length, 7.8 mm in diameter, and 2.3 g in weight [10]. The archival tags recorded ambient temperature, internal body temperature in the peritoneal cavity, light intensity, and swimming depth experienced by the tagged fish at 30-s intervals in this release survey. Sunrise, sunset, and daylight hours were calculated from the recorded light intensity data, and horizontal positions (latitude and longitude) were estimated at one point per day using an astronomy-based algorithm [44]. The estimated positions were corrected for latitude and longitude by comparing tag-recorded water temperature and swimming depth with SST and seafloor depth [45]. All procedures were conducted following ethical standards consistent with the current animal experimentation guidelines of the Japan Fisheries Research and Education Agency. The study area in this research was defined as 21–32°N, 121–134°E, covering waters from the Yangtze River estuary to the ECS. Of the 1,975 days of skipjack position data obtained, 1,627 data points falling within the study area were used for analysis (Fig 2b).

**Table 1:** Summary of skipjack tuna data collected during the release surveys around the Nansei Islands using archival tags.

	Release				Recapture				
Tag No.	Date	Latitude	Longitude	FL (cm)	Date	Latitude	Longitude	FL (cm)	Period (days)
468	2012/02/10	24.43°N	122.85°E	44.0	2012/04/19	29.55°N	130.40°E	—	69
864	2012/02/13	24.43°N	122.85°E	42.0	2012/03/22	25.33°N	124.97°E	—	38
967	2012/04/07	24.38°N	122.92°E	40.5	2012/06/30	24.63°N	123.85°E	—	84
977	2012/04/08	24.38°N	122.92°E	42.5	2012/05/11	30.00°N	129.75°E	—	33
1000	2012/04/12	24.37°N	122.97°E	40.5	2012/09/11	29.35°N	130.17°E	—	152
1039	2012/04/09	24.38°N	122.92°E	41.0	2012/06/08	30.33°N	130.33°E	45.2	60
2205	2014/03/10	24.42°N	122.87°E	39.0	2014/08/21	24.63°N	123.85°E	45.0	164
2774	2014/05/11	24.30°N	123.17°E	41.0	2014/09/16	29.47°N	129.57°E	44.0	128
2778	2014/05/12	24.30°N	123.17°E	43.0	2014/05/23	24.38°N	122.88°E	—	11
3402	2015/03/19	24.43°N	122.90°E	49.0	2015/04/02	24.38°N	122.88°E	52.0	14
3480	2015/05/30	24.32°N	122.82°E	44.0	2016/07/04	29.90°N	129.33°E	68.0	401
3532	2015/05/31	24.37°N	122.93°E	48.5	2015/08/06	32.90°N	139.42°E	51.0	67
4160	2016/04/13	24.32°N	122.82°E	40.5	2016/09/14	24.63°N	123.85°E	—	154
4355	2016/02/09	24.27°N	122.88°E	40.5	2016/10/08	28.00°N	129.00°E	48.5	242
4375	2016/02/09	24.27°N	122.88°E	44.0	2016/04/09	—	—	45.0	60
4551	2016/04/14	24.32°N	123.17°E	41.0	2016/07/01	30.33°N	130.50°E	41.2	78
4582	2016/04/14	24.32°N	123.17°E	41.0	2016/09/10	24.63°N	—	—	149
4614	2016/04/14	24.32°N	123.17°E	41.5	2016/06/24	29.45°N	129.58°E	51.0	71

**Fig 2:**
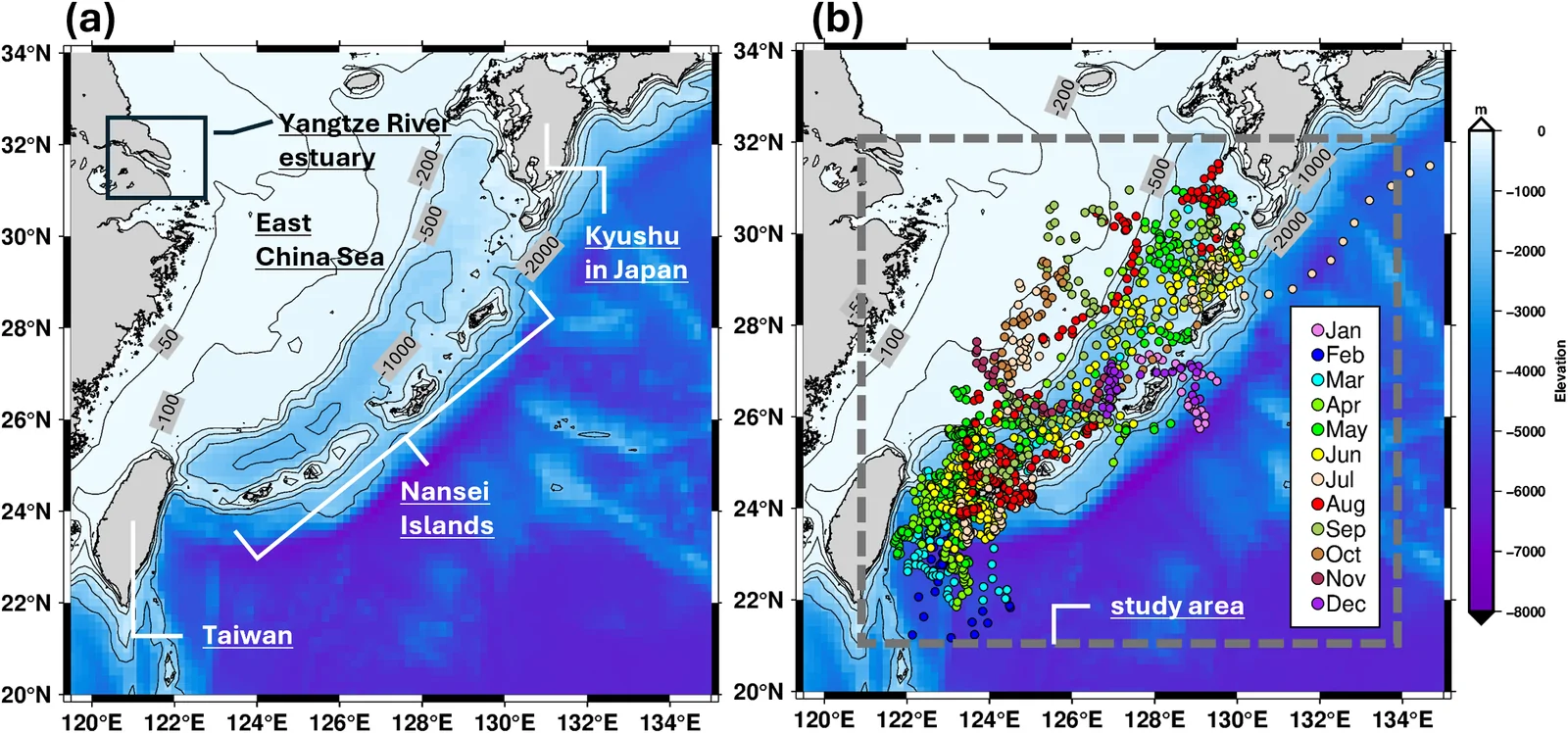
Study area and estimated locations of skipjack tuna. (a) Geographical information of the ECS. Numbers on contour lines indicate water depth in meters. (b) Study area (gray dashed line) and estimated positional data of skipjack tuna. The positions of skipjack tuna are shown in different colors for each month. Bathymetric data from the SRTM15 + global relief model [46].

### Environmental data

The data used included SST, SSS, SSC, and sea level anomaly (SLA), along with SST and SSS gradients (∇SST and ∇SSS) calculated from SST and SSS maps, respectively, as horizontal data (Table 2). The two gradients used the magnitudes (scalar) calculated from the vector difference of adjacent data points. For vertical data, temperature and salinity were obtained from Argo floats, and DO data from WOA 2023 were used.

**Table 2:** Environmental data list.

Variables	Product	Spatial resolution	Temporal resolution
SSS (psu)	Multi Observation Global Ocean Sea Surface Salinity and Sea Surface Density Product	0.125° × 0.125°	Daily
∇SSS (psu grid^−1^)	Calculated from SSS		Daily
SST (°C)	GHRSST Level 4 MUR 0.25° Global Foundation Sea Surface Temperature Analysis (v4.2)	0.25° × 0.25°	Daily
∇SST (°C grid^−1^)	Calculated from SST		Daily
SSC (mg m ^− 3^)	Global Ocean Color, Bio-Geo-Chemical, L4 from Satellite Observations	4 km × 4 km	Daily
SLA (m)	Global Ocean Gridded L 4 Sea Surface Heights And Derived Variables Reprocessed Copernicus Climate Service	0.25° × 0.25°	Daily
Salinity (psu), temperature (°C)	Argo float	—	10 days
DO (mL L ^− 1^)	World Ocean Atlas 2023: WOA2023	1° × 1°	Monthly climatology

SST has been widely used to characterize global tuna habitats [12], while SLA reveals oceanic mesoscale eddies, which play a crucial role in nutrient transport and primary productivity in oceans [47,48]. Freshwater from the Yangtze River affects SSS [49] and the stratification structure in the ECS, particularly contributing to the density difference between upper and lower waters during summer (Fig 3). This leads to the formation of hypoxic conditions in deeper waters [30]. Nutrient distributions are also closely related to the spread of low-salinity river water [33,50]. SSC is thus used as an indicator of the feeding environment for top predators, such as skipjack tuna [18]. ∇SST and ∇SSS represent abrupt changes in SST and SSS, respectively, with strong gradients marking the boundary between Yangtze-River-influenced low-salinity waters and the Kuroshio-influenced high-salinity waters near the Nansei Islands.

**Fig 3:**
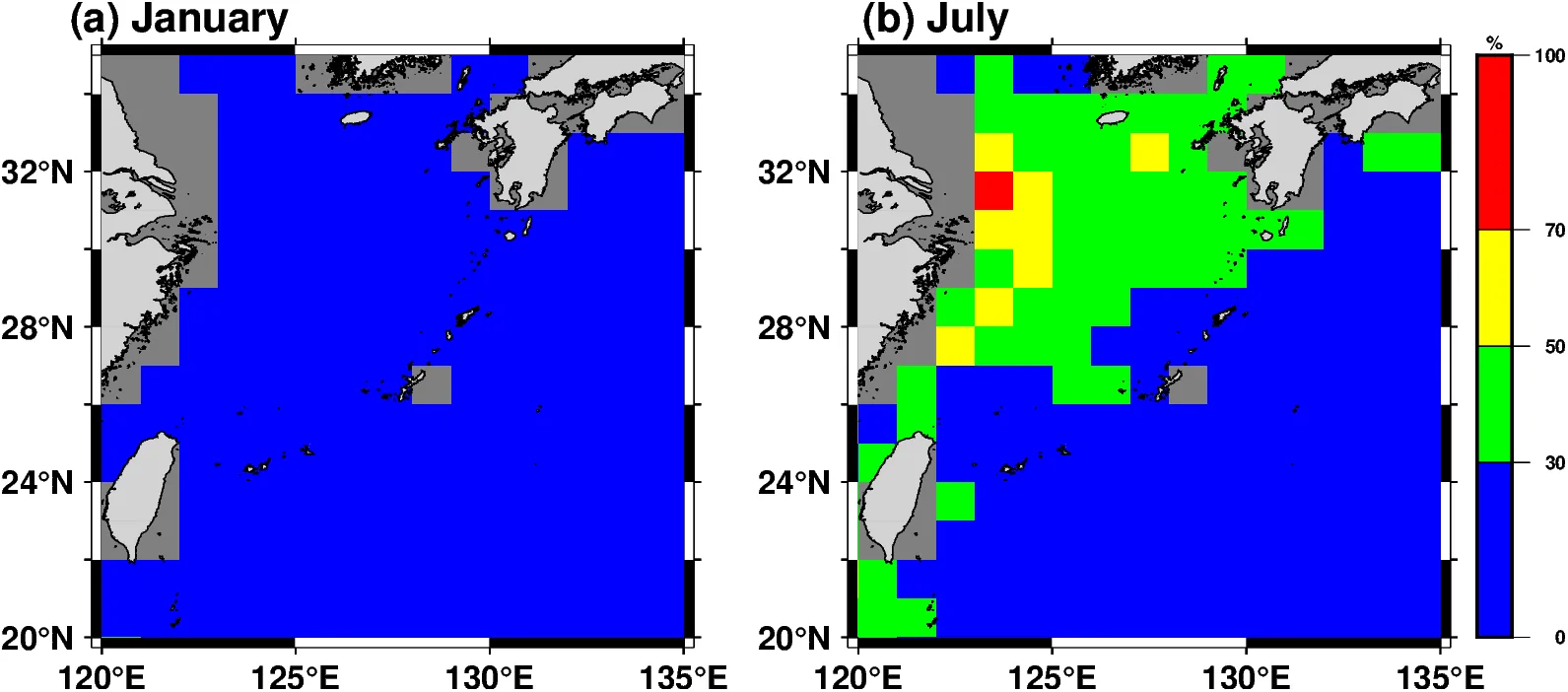
Contribution rate (%) of salinity changes to density differences between the sea surface and 40-m depth in (a) January and (b) July. Density differences between the surface and the 40-m depth were calculated using WOA (World Ocean Atlas 2023) temperature and salinity data (monthly climatology), and the proportion attributable to salinity changes was computed and visualized.

To investigate the sea surface environments experienced by skipjack tuna, mean values were extracted from 0.5° mesh data centered on each estimated position. Based on the resulting distributions, sea surface habitat conditions were examined from the Nansei Islands to the ECS. For vertical habitat analysis, temperature and salinity data from Argo floats were extracted to correspond with the vertical distribution of skipjack tuna. Argo float data available in the study area were identified using OceanOPS (https://www.ocean-ops.org/board?t=argo, accessed 2025/05/26) and subsequently downloaded. The analysis proceeded as follows. First, Argo float data with the shortest distance to the estimated skipjack positions were selected in pairs. A threshold range from 10 to 300 km was set in 1-km increments, and the distance at which significant discrepancies occurred between the Argo float and sea surface data was defined as the threshold. From pairs within this distance, temperature and salinity at depths corresponding to the daily mean swimming depth of skipjack tuna were extracted, and statistical values were calculated from these data. However, as Argo floats are rarely deployed in the ECS and its surrounding continental shelf regions, the present analysis focuses on characterizing the vertical environmental conditions experienced by skipjack tuna in the waters around the Nansei Islands.

Skipjack tag data were utilized to focus on skipjacks that experienced low-salinity waters. The skipjack tuna studied herein were distributed from around the Nansei Islands to the ECS. Therefore, those that experienced SSS values below the 95th percentile of the extracted SSS data were selected to examine responses in low-salinity waters. The reason for using statistical measures rather than specific numerical values as a standard is that, although salinity is important for fish and plays a role in osmoregulation [24,25], there are no numerical standards for skipjack tuna’s salinity tolerance. The migration routes of these selected skipjack tuna were mapped to investigate horizontal associations with sea surface environments. In addition, statistical values for individuals migrating in low- and normal-salinity waters were calculated. Furthermore, the vertical DO and temperature experienced by individuals in low-salinity waters were analyzed. Because the ECS was relatively shallow and Argo floats were not widely deployed in this region, three-dimensional monthly climatological data from WOA were used for this analysis. The depth distributions of skipjack in both low- and normal-salinity waters were analyzed, and the DO and ambient temperature recorded by the archival tags at corresponding depths were examined. It should be noted that the WOA DO data represent monthly climatological averages and therefore do not capture day-to-day variability in dissolved oxygen concentrations. Seasonal differences in the environmental conditions experienced by skipjack tuna were examined. A bootstrap resampling approach was applied to account for the unequal number of observations between seasons. The larger dataset was repeatedly subsampled without replacement (n = 10,000 iterations) to match the sample size of the smaller dataset, and seasonal differences were assessed based on the resulting resampled distributions.

### Estimation of energy intake

Because the ECS is characterized by high primary production driven by the nutrient load from the Yangtze River, feeding opportunities for skipjack tuna may have increased in this region. Therefore, daily energy intake was estimated for individuals using the heat increment of feeding (HIF) to investigate changes in energy intake within the ECS. HIF represents increased metabolic activity resulting from ingestion, digestion, absorption, and assimilation [39,40,51], leading to increased oxygen consumption and associated heat production [52]. The use of HIF for estimating skipjack energy intake has been demonstrated previously [39]. Here, skipjack energy intake was estimated using a previously established methodology. To estimate HIF, a baseline body temperature (*T*_b_), assuming nonfeeding periods, was required. This baseline was calculated using a heat balance model that accounted for fish body temperature as a function of ambient temperature and movement [53]:


$$\begin{array}{c} \frac{dT_{\text{b}}}{dt}\;=\;\lambda \;\left(T_{\text{a}}\;-\;T_{\text{b}}\;\right)\;+\;T_{\text{m}}. \end{array}$$ (1)

The time variation rate of body temperature depended on the difference between the ambient temperature (*T*_a_) and body temperature (*T*_b_), the whole-body heat transfer coefficient (*λ*), and the rate of temperature change due to metabolic heat production (*T*_m_). Using these parameters, body temperature during nonfeeding periods was estimated. HIF was defined as the integral of the temperature difference when the measured body temperature exceeds the estimated nonfeeding temperature from the starting time (*t*_s_) to the return time (*t*_f_):


$$\begin{array}{c} \varphi \;=\;\int_{t_{s}}^{t_{\text{f}}}(T_{b}\;-\;{\hat{T}}_{b}\;)\;dt, \end{array}$$ (2)

where ${\hat{T}}_{\text{b}}$ is the body temperature estimated using Equation (1).

In this study, archival tag data were used to obtain *T*_b_ and *T*_a_. Meanwhile, *λ* and *T*_m_ were estimated using nonlinear least squares optimization (for details, see Aoki *et al*. [39]). The previously reported positive correlation between estimated HIF and food caloric intake (regression coefficient: 1.47 [39]) was used to calculate the energy intake. This regression coefficient has previously been applied in the northwestern Pacific Ocean [39] and is therefore considered applicable to the present study conducted in the same region. Statistical values of the estimated energy intake were computed.

## Results

### Environmental associations of skipjack tuna around the ECS

The surface environments experienced by skipjack ranged from 31.3 to 35.2 psu for SSS, 18.1°C–31.3°C for SST, 0.0 to 1.7 mg m^−3^ for SSC, −0.6 to 0.4 m for SLA, 0.0 to 0.6 psu grid^−1^ for ∇SSS, and 0.0°C–1.9°C grid^−1^ for ∇SST (Fig 4 and Tables 3–4). No significant differences were observed between SST and Argo float temperature up to 300 km (S1 Fig). However, significant differences were found between archival tag ambient temperature and Argo float temperature beyond 294 km and between SSS and Argo float salinity beyond 158 km (S1 Fig). The total number of days with data within these thresholds was 510 days for temperature and 163 days for salinity. Pairs within 177 km were concentrated east of Taiwan, with no pairs in the ECS (S2 Fig). The distributions of the extracted salinity and temperature data under these thresholds are shown in Fig 5. High-salinity and warm-water distributions similar to sea surface distributions were observed in the Argo float data (mean values: salinity 34.8 ± 0.2 psu; temperature: 24.7° ± 2.2°C).

**Table 3:** Statistics of extracted data. Environmental data (SSS, SST, SSC, SLA, ∇SSS, and ∇SST) were extracted from 0.5° mesh data around the estimated skipjack positions for all 18 individuals.

	SSS (psu)	SST(°C)	SSC (mg m ^− 3^)	SLA (m)	∇SSS (psu grid^−1^)	∇SST (°C grid^−1^)
Mean	34.4	26.8	0.2	0.0	0.1	0.3
Median	34.5	27.2	0.1	0.0	0.0	0.2
SD	0.5	2.5	0.1	0.2	0.0	0.2
min	31.3	18.1	0.0	−0.6	0.0	0.0
max	35.2	31.3	1.7	0.4	0.6	1.9
5th percentile	33.5	22.7	0.1	−0.3	0.0	0.1
95th percentile	34.9	30.5	0.4	0.2	0.1	0.8

**Table 4:** Statistics of extracted data. Environmental data (SSS, SST, SSC) were extracted from 0.5° mesh data around the estimated skipjack positions for each of the 18 individuals.

	SSS (psu)			SST (°C)			SSC (m m^-3^)		
**Tag** **No.**	**Mean** **±SD**	**5%**	**95%**	**Mean** **±SD**	**5%**	**95%**	**Mean** **±SD**	**5%**	**95%**
468	34.6 ± 0.1	34.3	34.8	22.3 ± 1.7	18.9	24.5	0.3 ± 0.3	0.1	0.8
864	34.6 ± 0.1	34.4	34.7	23.8 ± 0.8	22.5	25.0	0.2 ± 0.1	0.1	0.3
967	34.3 ± 0.2	34.0	34.6	26.7 ± 1.4	24.3	28.4	0.1 ± 0.1	0.0	0.2
977	34.1 ± 0.5	33.2	34.7	23.7 ± 1.4	21.1	25.5	0.3 ± 0.2	0.2	0.5
1000	33.8 ± 0.5	32.9	34.5	27.0 ± 2.0	23.6	29.8	0.1 ± 0.0	0.1	0.2
1039	34.2 ± 0.4	33.4	34.7	24.3 ± 1.5	21.5	26.1	0.2 ± 0.1	0.1	0.4
2205	34.5 ± 0.2	34.3	35.0	25.6 ± 1.7	22.4	28.2	0.1 ± 0.0	0.1	0.2
2774	34.3 ± 0.2	33.8	34.7	28.9 ± 1.2	26.4	30.5	0.1 ± 0.0	0.1	0.1
2778	34.5 ± 0.1	34.4	34.7	26.4 ± 0.4	26.0	27.1	0.1 ± 0.0	0.1	0.1
3402	34.6 ± 0.2	34.3	34.8	24.7 ± 0.4	24.3	25.6	0.2 ± 0.0	0.1	0.2
3480	34.1 ± 0.7	32.7	34.9	26.5 ± 2.2	22.8	29.7	0.3 ± 0.2	0.1	0.8
3532	34.4 ± 0.2	34.1	34.8	28.2 ± 0.6	27.2	28.9	0.1 ± 0.0	0.1	0.1
4160	34.5 ± 0.2	34.3	34.8	27.1 ± 0.5	26.1	27.6	0.1 ± 0.0	0.1	0.2
4355	34.6 ± 0.2	34.1	34.9	27.9 ± 2.5	23.7	30.9	0.1 ± 0.0	0.0	0.1
4375	34.9 ± 0.1	34.8	35.0	23.7 ± 0.5	23.0	24.5	0.2 ± 0.0	0.1	0.2
4551	34.5 ± 0.2	34.2	34.8	27.9 ± 0.7	26.8	29.1	0.1 ± 0.0	0.1	0.1
4582	34.5 ± 0.2	34.2	34.8	29.3 ± 1.4	26.8	31.1	0.1 ± 0.0	0.0	0.1
4614	34.4 ± 0.3	33.8	34.7	27.0 ± 1.6	23.1	28.7	0.1 ± 0.1	0.1	0.4
									
All	34.4 ± 0.5	33.5	34.9	26.8 ± 2.5	22.7	30.5	0.2 ± 0.1	0.1	0.4

**Fig 4:**
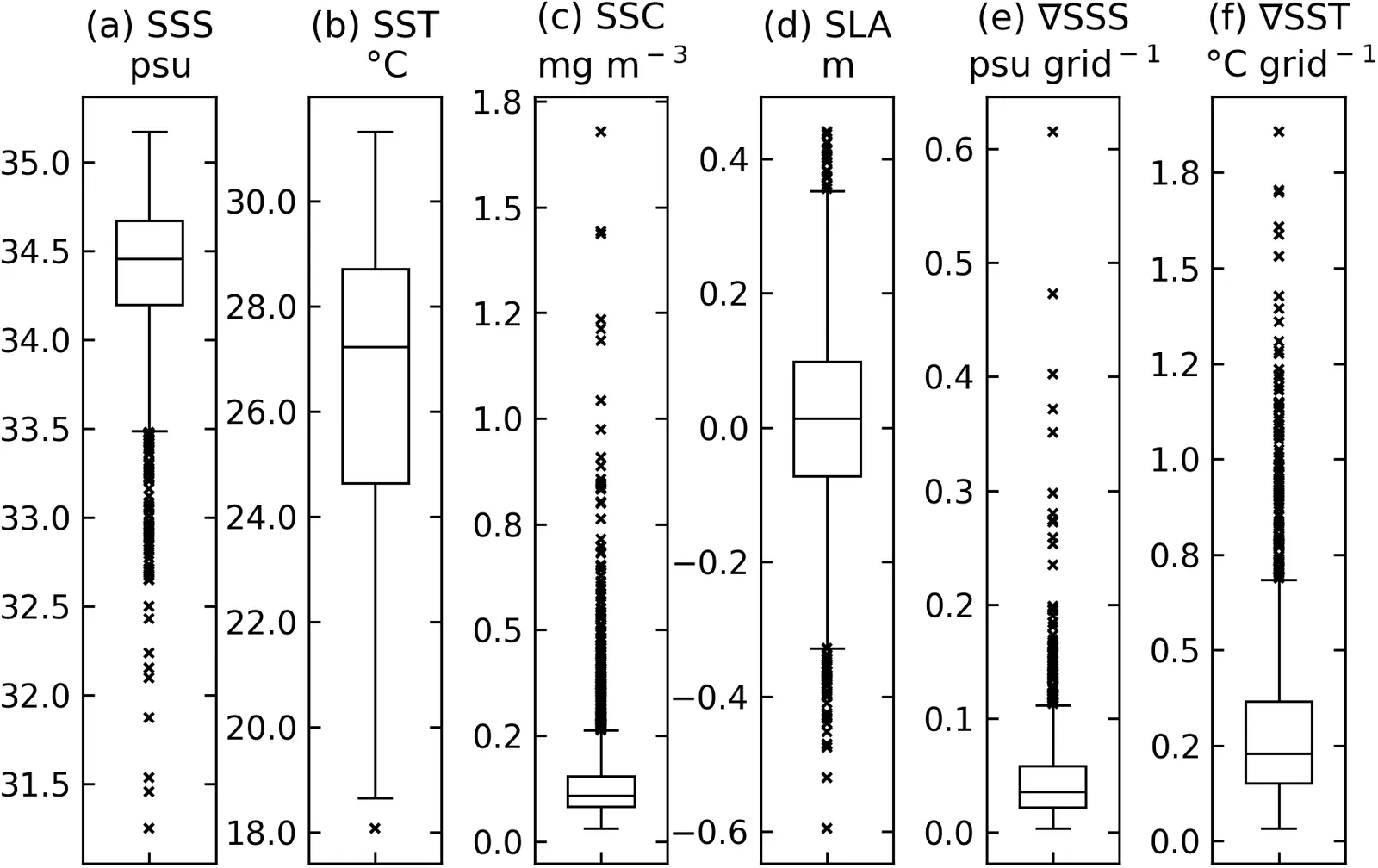
Box-and-whisker plots of physical parameters experienced by skipjack tuna based on satellite-derived environmental data. Whiskers extend to the most extreme data points within 1.5× interquartile range (IQR) from the quartiles. Data points beyond the whiskers are plotted individually as outliers and marked with crosses (×). Satellite data are used as environmental data. (a) SSS, (b) SST, (c) SSC, (d) SLA, (e) ∇SSS, and (f) ∇SST.

**Fig 5:**
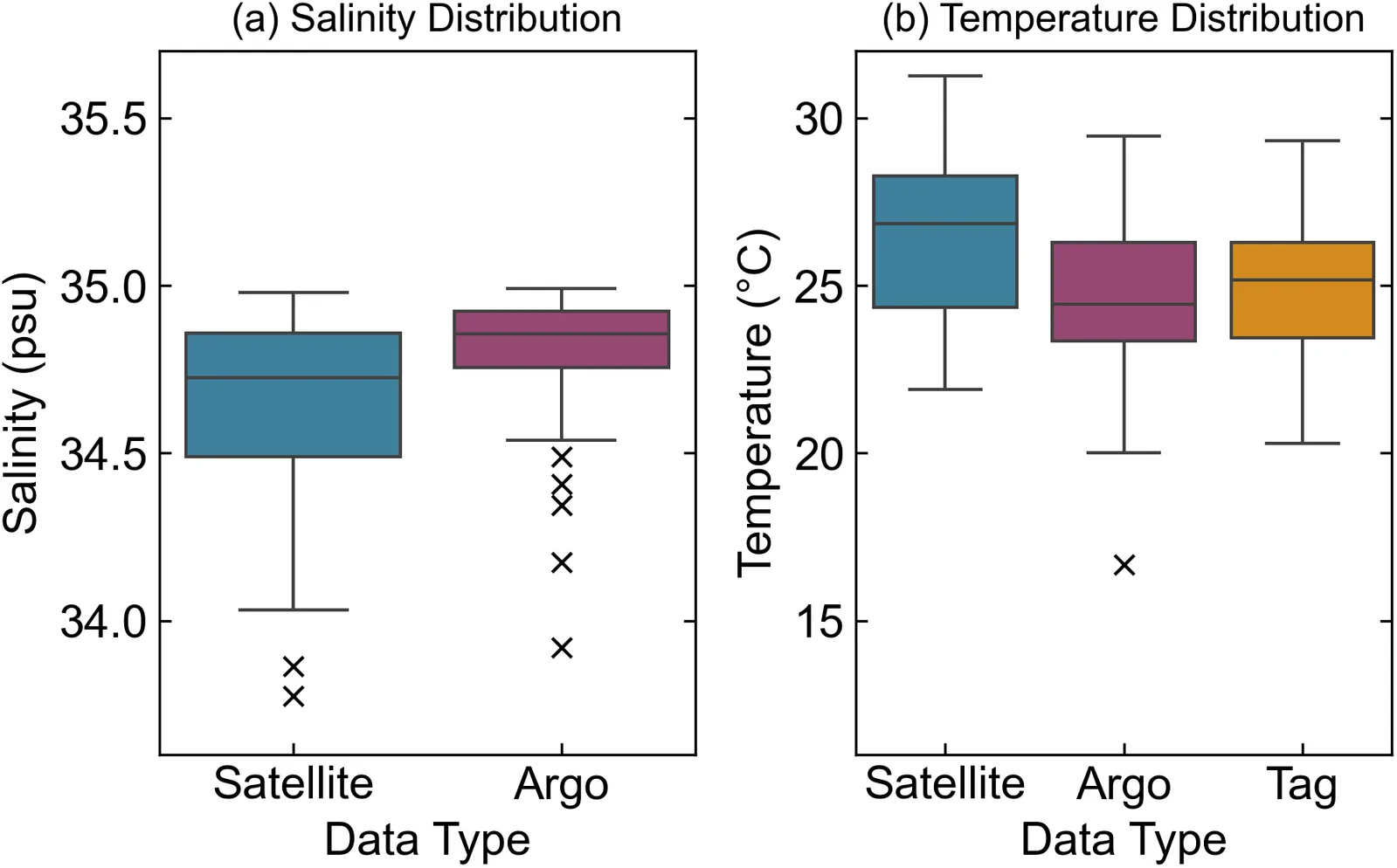
Box-and-whisker plots of satellite-derived data (salinity and temperature), Argo float data (salinity and temperature), and archival tag data (ambient temperature) extracted using thresholds. (a) Salinity; (b) temperature. Whiskers extend to the most extreme data points within 1.5 × IQR from the quartiles. Data points beyond the whiskers are plotted individually as outliers and marked with crosses (×). Salinity figures from archival tag data are not shown because salinity sensors were not installed on the archival tags.

Based on Fig 4, the 95th percentile of SSS experienced by skipjack (≈33.5 psu, S3 Fig) was defined as the threshold. Individuals that experienced salinity values below this threshold were extracted (Table 5). A total of five individuals were identified—three in spring and two in summer. Their estimated positions are shown along with SSS in Fig 6. These individuals were concentrated on the northwest side of the Nansei Islands, where SSS gradually decreased toward the continental margin of the ECS.

**Table 5:** List of skipjack individuals that entered low-salinity water areas.

	Season	Tag No.	Entry to SSS < 33.5 psu	Retreat from SSS < 33.5 psu
Su1	Summer	3480	2015/07/11	2015/10/11
Su2	Summer	1000	2012/07/09	2012/09/05
Sp1	Spring	977	2012/04/21	2012/04/27
Sp2	Spring	1039	2012/04/23	2012/05/05
Sp3	Spring	4614	2016/05/05	2016/05/05

**Fig 6:**
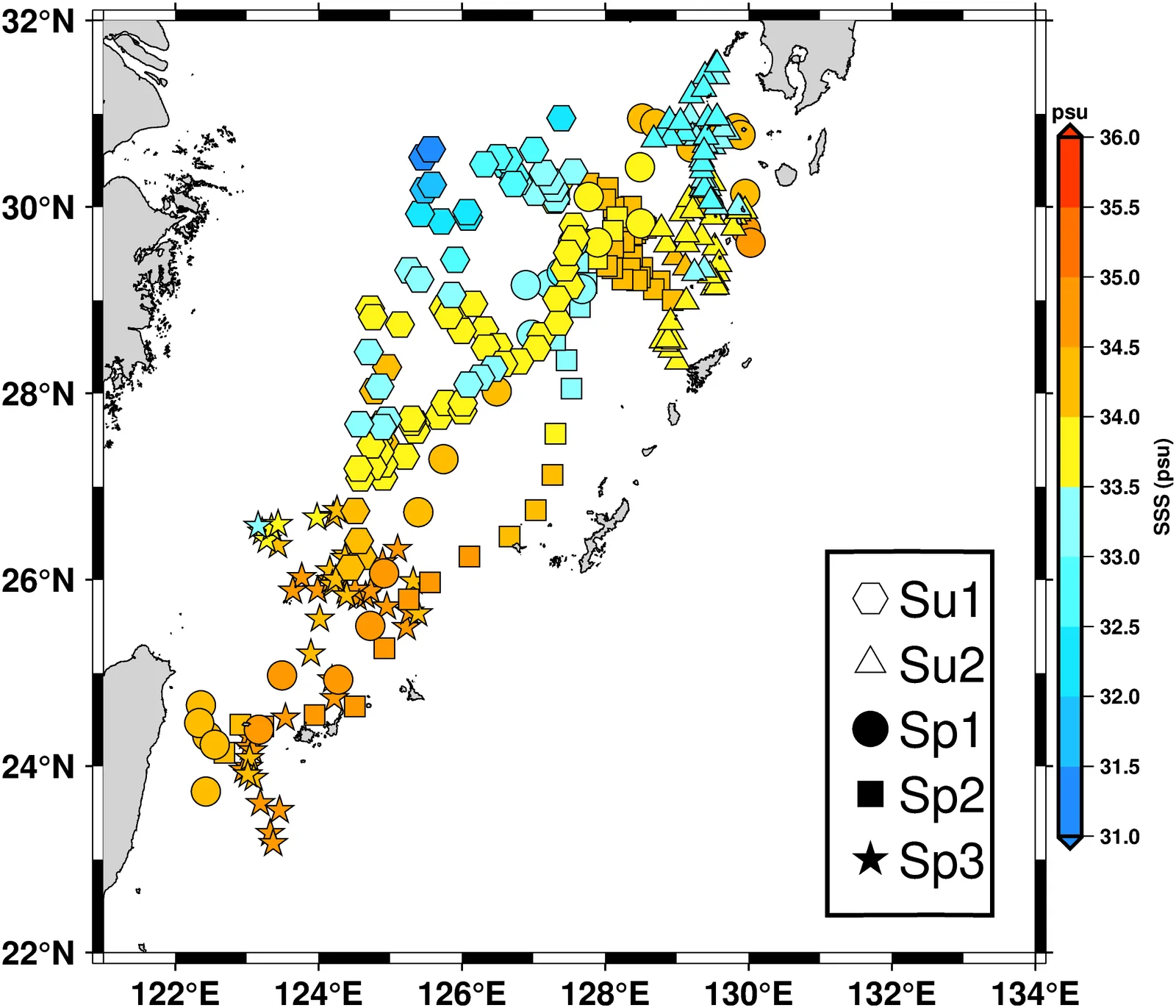
Salinity at the locations of low-salinity-exposed individuals. Data were extracted from July to September for summer entries and from April to May for spring entries.

The estimated locations of the five tracked individuals were mapped with sea surface data corresponding to periods of lowest salinity (Fig 7). During summer, they moved toward ~30.5°N and 125.5°E (Fig 7a). Su1 traveled from the central ECS southwestward through low-salinity waters extending from the northwest (9/6–9/9) and then shifted northwest (9/10–9/14). Subsequently, it entered a low-salinity zone (minimum of 31.3 psu) and moved southward (9/14–9/17). The water temperature in this region exceeded ~25°C (Fig 7e). Throughout this season, strong ∇SSS and ∇SST were not evident (Figs 7q and 7u), and skipjack positions were concentrated in areas with high SSC (~1.2 mg m^−3^: Fig 7i). Su2, like Su1, entered the ECS after migrating northward to waters off the Kyushu coast (Fig 7b). It appeared to follow regions where SLA values hovered around 0 m during its northward movement (Fig 7n). Additionally, SST across the ECS remained uniform around 28°C (Fig 7f), and no strong ∇SSS or ∇SST patterns were observed (Figs 7r and 7v), as with Su1.

**Fig 7:**
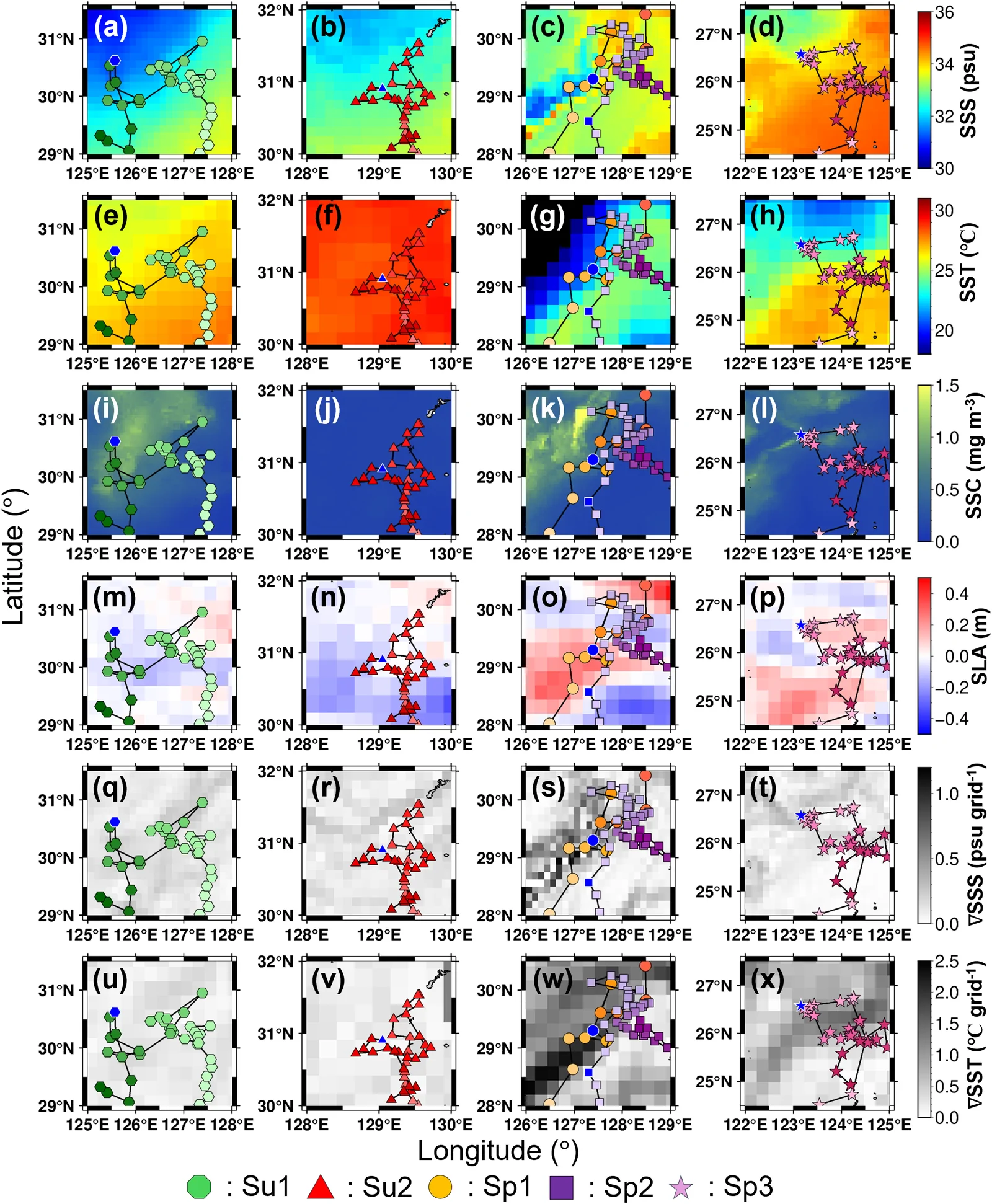
Mapped environmental data and estimated skipjack tuna locations. Blue markers indicate the day of lowest salinity for each individual. From top to bottom: SSS, SST, SSC, SLA, ∇SSS, and ∇SST. Sp1 and Sp2 were observed during the same period and are shown on the same map. The environmental data shown represent the day on which each individual experienced the lowest SSS (Su1: 2015/9/14; Su2: 2012/8/24; Sp1 and Sp2: 2012/4/25; Sp3: 2016/5/5). Color intensity represents time progression (light to dark).

For Sp1 and Sp2, strong ∇SSS and ∇SST were observed in the ECS during spring season, and the estimated positions were clustered near the frontal zone northwest of the Nansei Islands (Figs 7s and 7w). These low-salinity waters originated not only from the Yangtze River but also from the coastal region of Zhejiang Province, China, forming a tongue-like extension to the northwest of the Nansei Islands (Fig 7c). The minimum SSS experienced by these individuals was 32.8 psu. SST showed a similar distribution, with waters below ~18°C spreading across the ECS (Fig 7g). In contrast, the estimated positions of Sp1 and Sp2 indicated that they did not enter colder zones (minimum of 18.1°C) across strong ∇SST (~2.0°C grid^−1^), instead remaining near regions of high SSC (Fig 7k). Similarly, Sp3 entered the ECS from northeast of Taiwan and lingered near areas with pronounced ∇SST (1.5°C grid^−1^) and elevated SSC (~1.5 mg m^−3^; Figs 7x and 7l). Additionally, Sp3 appeared to follow regions with SLA values close to 0 m upon entry into the ECS (~1.0 mg m^−3^; Fig 7p).

### Movement differences of skipjack tuna in the ECS

Environmental data from skipjack tuna that entered the ECS were categorized using a salinity threshold of 33.5 psu, and mean values ± standard deviations (SD) were calculated (Table 6). Due to differences in the number of data points, we did not perform a statistical test here. In the summer group, SST showed similar values between high- and low-salinity areas, whereas the spring group experienced lower SST in low-salinity waters. Additionally, the tracked individuals encountered high SSC and SLA values near 0 m while residing in low-salinity zones. Regarding ∇SSS and ∇SST, the spring group tended to migrate through regions with elevated ∇SST, whereas marked changes in ∇SSS and ∇SST were not apparent in the summer group during ECS entry.

**Table 6:** Mean ocean environmental conditions experienced by skipjack tuna, grouped by sea surface salinity (SSS) threshold (33.5 psu) and season. Sea surface environmental data were extracted according to estimated positions between July and September (summer individuals) and between April and May (spring individuals). Data were grouped using a salinity threshold of 33.5 psu and are presented as mean ± SD.

Season	Spring		Summer	
**Area**	**< 33.5 psu**	**≥33.5 psu**	**<33.5 psu**	**≥33.5 psu**
N	14	153	74	91
SSS (psu)	33.3 ± 0.2	34.3 ± 0.3	33.0 ± 0.5	33.8 ± 0.2
SST (°C)	22.8 ± 1.9	25.1 ± 1.9	28.4 ± 1.1	28.4 ± 1.0
SSC (mg m ^− 3^)	0.4 ± 0.2	0.2 ± 0.1	0.3 ± 0.3	0.3 ± 0.3
SLA (m)	0.1 ± 0.1	0.0 ± 0.1	−0.0 ± 0.1	−0.0 ± 0.1
∇SSS (psu grid^−1^)	0.2 ± 0.2	0.1 ± 0.1	0.1 ± 0.1	0.1 ± 0.0
∇SST (°C grid^−1^)	1.1 ± 0.5	0.6 ± 0.4	0.2 ± 0.1	0.2 ± 0.1

Daytime depth distributions were examined for the five individuals in low-salinity waters and in the open ocean, when skipjack typically swim across a wide depth range (Fig 8). The data were classified according to whether the estimated positions were on the continental shelf (bathymetry < 200 m) with salinity below 33.5 psu or in deeper waters (bathymetry > 200 m). Daytime periods were identified using light intensity and timestamp data from archival tags. The 95th percentile depth during the day was defined as the main swimming depth, which was 67.1 m in low-salinity waters and 118.2 m in the open ocean for the spring group (Fig 8a). For the summer group, the corresponding depths were 66.8 m in low-salinity waters and 106.9 m in the open ocean (Fig 8b). In both seasons, the swimming depth was shallower in low-salinity waters (i.e., on the continental shelf) than in the open ocean. Additionally, individual-level Spearman rank correlations between swimming depth and SSS were calculated for each of the five focal individuals (Table 7). Both summer individuals showed significant positive correlations between swimming depth and SSS. Among the spring individuals, Sp1 showed a significant negative correlation, Sp3 showed a significant positive correlation, and Sp2 showed no significant correlation.

**Table 7:** Spearman rank correlation coefficients between skipjack tuna daytime 5th percentile depth and SSS. Normality was assessed using the Shapiro–Wilk test. As normality was rejected for several individuals, Spearman’s rank correlation coefficient was applied as a non-parametric alternative.

SKJ No.	correlation	p value
Su1	0.65	< 0.001
Su2	0.34	< 0.001
Sp1	−0.40	0.020
Sp2	0.16	0.209
Sp3	0.38	0.001

**Fig 8:**
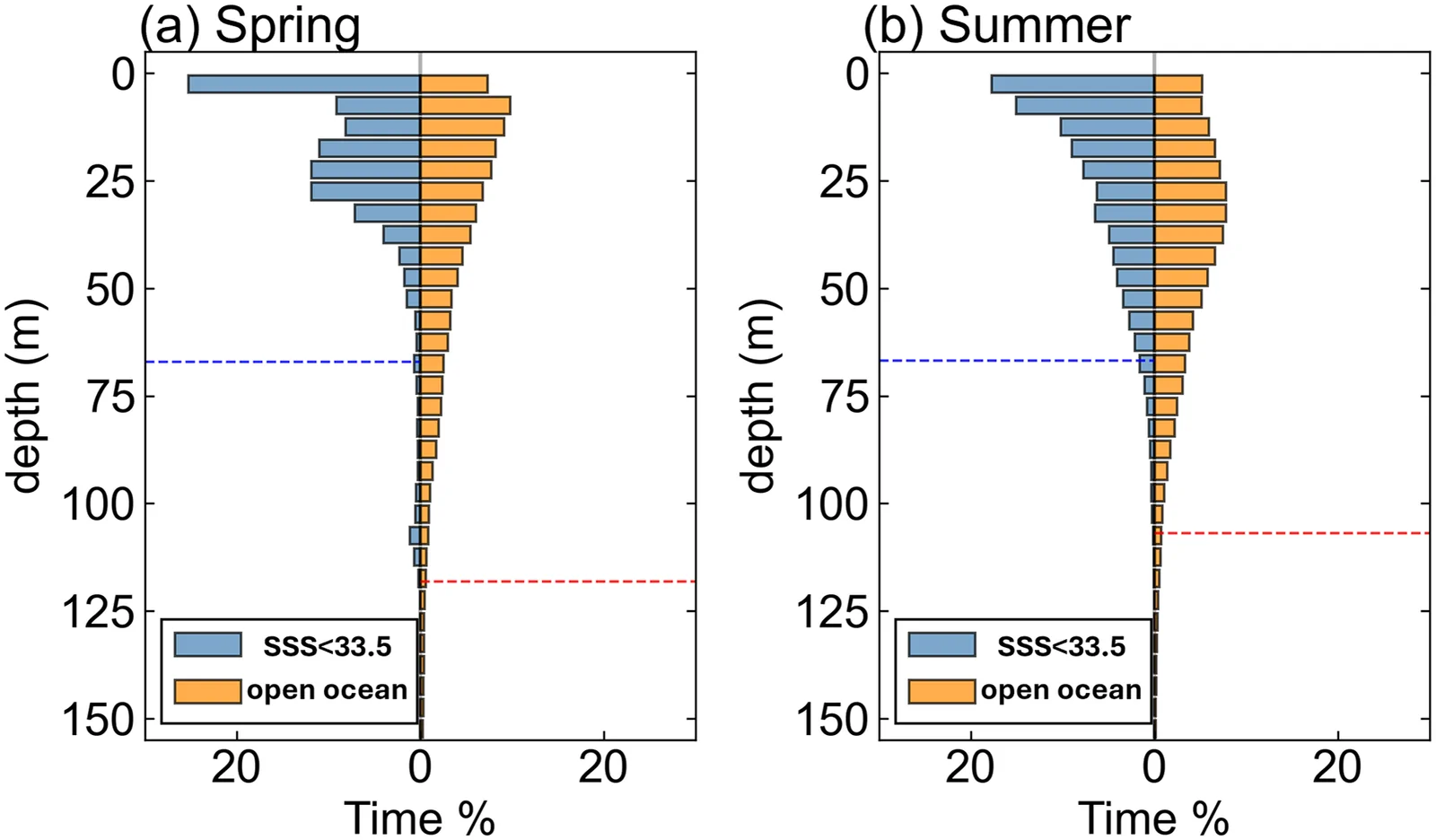
Depth distribution of skipjack tuna in low-salinity and open-ocean areas during daytime: (a) spring group; (b) summer group. The low-salinity environment was defined as <33.5 psu, and the open-ocean environment was defined as a bathymetry of >200 m. Dotted lines indicate the 95th percentile values: spring, low-salinity = 67.1 m; spring, open ocean = 118.2 m; summer, low-salinity = 66.8 m; summer, open ocean = 106.9 m.

The vertical distributions of ambient temperature (from the archival tags) and DO from WOA monthly climatological data for each salinity area are shown in Fig 9. In the spring group (Fig 9a), the temperature remained relatively uniform down to ~90 m in the open ocean because of vertical mixing. In contrast, in low-salinity waters, the mixed layer extended to ~40 m; the temperature gradually declined below this depth, indicating a shallower stratification compared with that in the open ocean. For the summer group, thermal stratification was present in both salinity areas, with only slight temperature differences observed (Fig 9b). Meanwhile, the DO distributions in the open ocean were relatively uniform down to ~100 m for the spring group (Fig 9c), with concentrations remaining above ~4.5 mL L^−1^, albeit with some vertical variation. In contrast, at SSS < 33.5 psu, surface DO was higher, exceeding 5.5 mL L^−1^ (Fig 9c). In low-salinity waters, DO began to decline below 40 m and reached below 4.0 mL L^−1^ at ~50 m for the summer group (Fig 9d). The 95th percentile ambient temperature and DO at the 95th percentile swimming depth in low-salinity waters were 18.0°C and 4.5 mL L^−1^ for the spring group and 20.5°C and 3.6 mL L^−1^ for the summer group, respectively.

**Fig 9:**
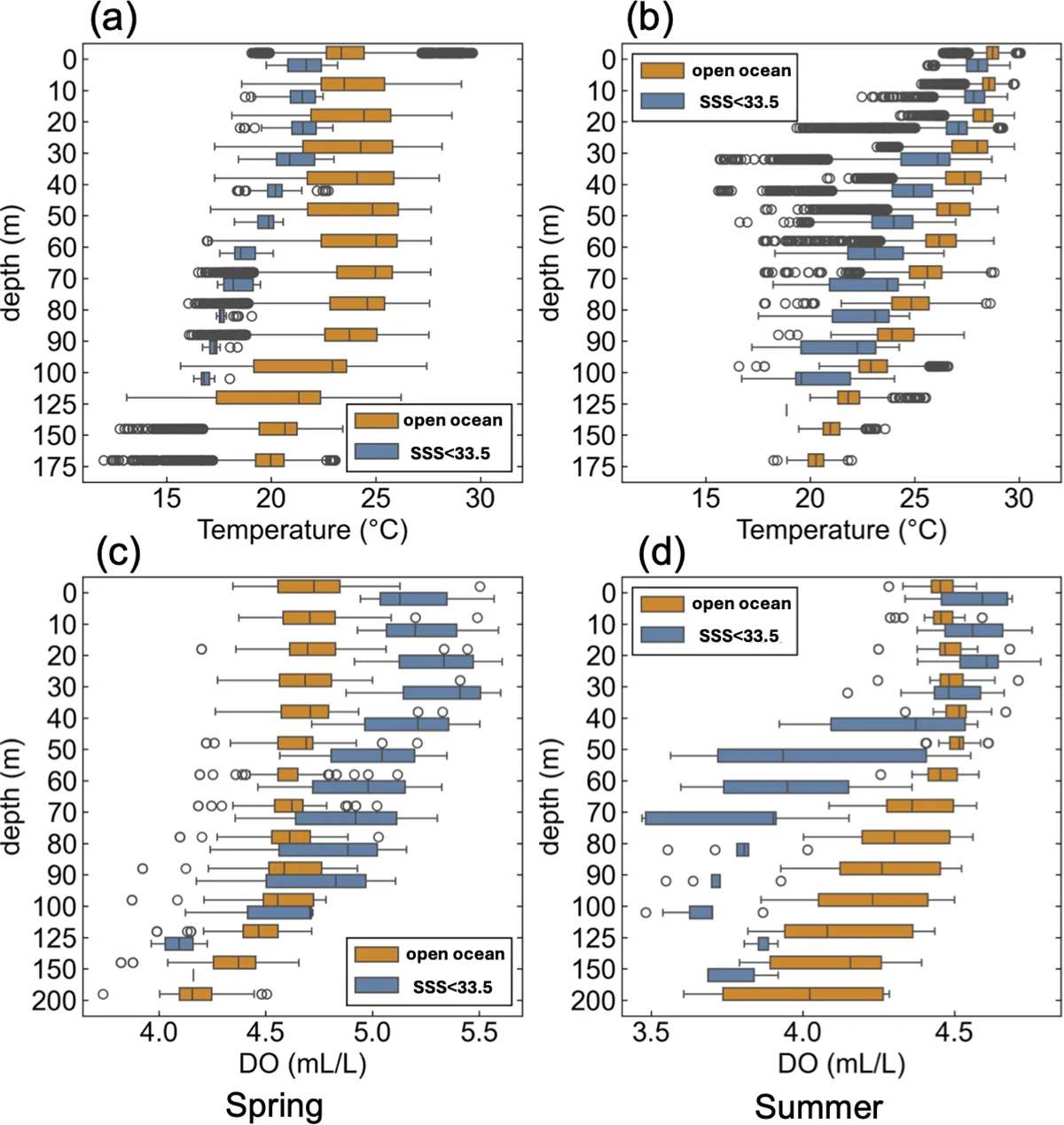
Vertical structures at the location of skipjack tuna in low-salinity and open-ocean areas. Panels (a) and (b) show ambient temperature recorded by the archival tags; panels (c) and (d) show DO profiles from WOA data. Ambient temperature and DO by depth are presented as box plots. Whiskers extend to the most extreme data points within 1.5 × IQR from the quartiles. Data points beyond the whiskers are plotted individually as outliers and marked with circles. Panels (a) and (c) show the results for spring. Panels (b) and (d) show the results for summer.

Pairwise Spearman correlation analyses were conducted between SSS and co-varying environmental variables (SST, SSC, and DO) for each of the five focal individuals to identify variables significantly cross-correlated with salinity (Fig 10). The results revealed seasonal differences in the correlations between SSS and environmental variables. Among the Spring individuals, Sp1 and Sp2 showed significant positive correlations between SSS and SST, whereas summer individuals (Su1 and Su2) showed significant negative correlations. SSC showed significant negative correlations with SSS across all individuals. For DO, spring individuals with the exception of Sp3 showed a significant positive correlation with either the daytime 5th percentile depth DO or the daytime mean depth DO, while both summer individuals showed significant positive correlations with both metrics. Bootstrap resampling analyses revealed consistent seasonal differences in the environmental conditions experienced by skipjack tuna (Fig 11). Dissolved oxygen concentrations were lower in summer than in spring at both the daytime mean depth and the daytime 5th percentile depth. Conversely, ambient water temperature at depth was lower in spring than in summer.

**Fig 10:**
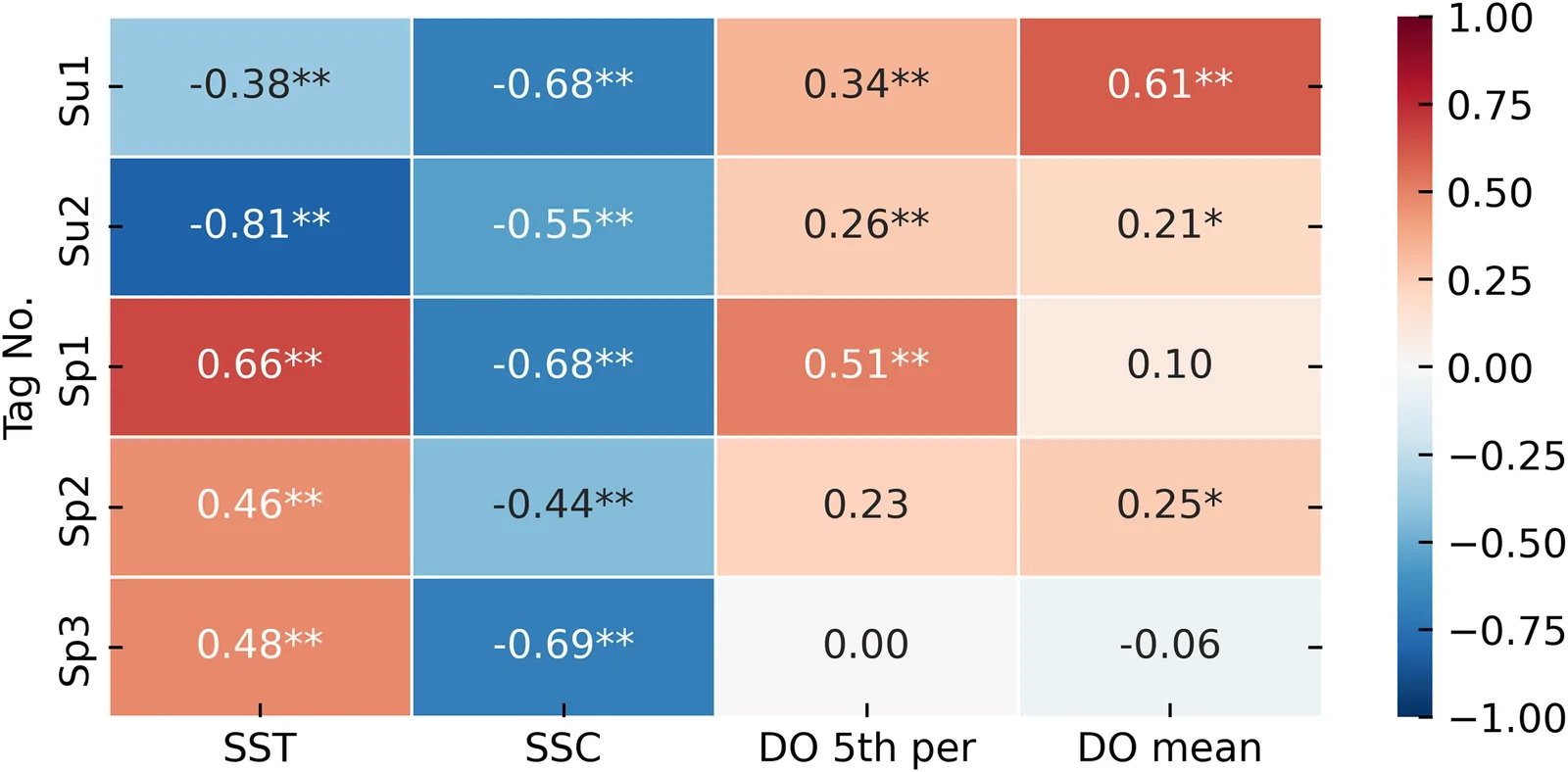
Spearman correlation coefficients between SSS and selected environmental variables (SST, SSC, daytime 5th percentile DO, and daytime mean DO) for the five individuals that entered low-salinity waters in the ECS. Single and double asterisks indicate p < 0.05 and p < 0.01, respectively. Normality was assessed using the Shapiro–Wilk test. As normality could not be confirmed for several variables, Spearman’s rank correlation coefficient was applied as a non-parametric alternative.

**Fig 11:**
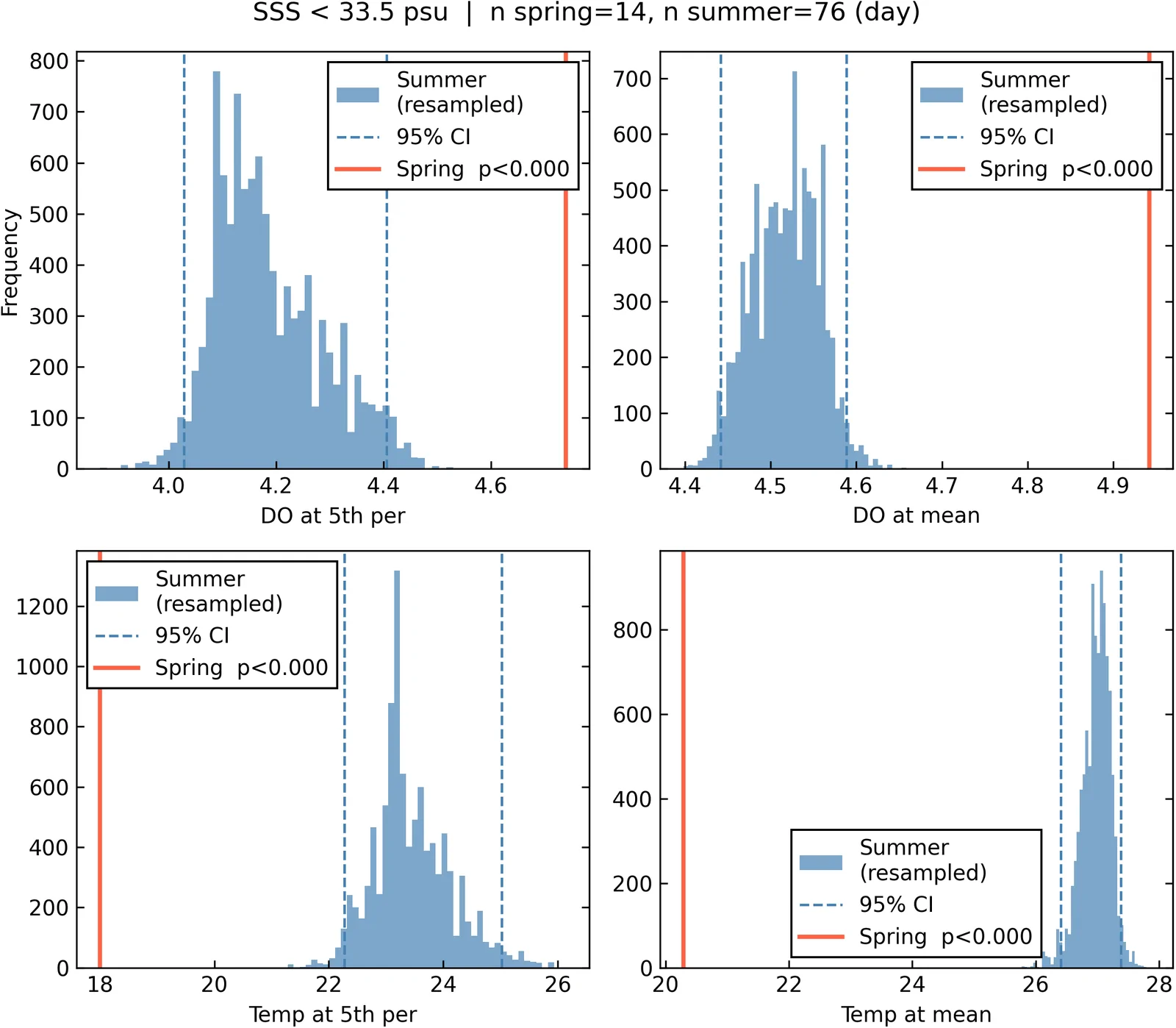
Bootstrap analysis comparing DO and ambient temperature recorded by archival tags between spring and resampled summer under low-salinity conditions (SSS < 33.5 psu; n = 14 days in spring, n = 76 days in summer). Median values were extracted at the daytime 5th percentile depth (left panels) and daytime mean depth (right panels) for each individual skipjack tuna. Blue histograms show the distribution of bootstrap-resampled median values in summer (10,000 iterations). Blue dashed lines indicate the 2.5th and 97.5th percentiles of the resampled distribution (95% confidence interval). Red lines indicate the observed median values in spring. All comparisons yielded p < 0.001, indicating significant seasonal differences in both DO and temperature.

### Estimation of energy intake

HIF was estimated from body temperature changes for the five individuals that entered the ECS, and daily energy intake was calculated from the HIF values. To convert HIF to energy intake, the regression coefficient (=1.47 [39]) mentioned in the “Materials and Methods” section was applied. Daily energy intake was then categorized according to SSS distributions and visualized using box plots (Fig 12). Energy intake tended to be higher in low-salinity waters (SSS < 33.5 psu) than in high-salinity waters (SSS > 75th percentile). However, individual-level Mann-Whitney U tests revealed inconsistent patterns across individuals: Sp1 and Su2 showed higher HIF values in low-salinity waters (p = 0.050 and p < 0.01, respectively), while Sp2 and Su1 showed no significant difference, with slightly higher values observed in high-salinity waters (Table 8).

**Table 8:** Mean ± standard deviation of daily energy intake of each skipjack tuna in relation to SSS. “Direction” indicates which salinity zone (low-salinity: SSS < 33.5 psu, or high-salinity: SSS ≥ 33.5 psu) showed higher estimated energy intake for each individual. Individual Sp3 has been omitted because there is only one day’s worth of data for low salinity conditions.

		Mean±SD				
SKJ No.	n [low, high]	low	high	U stat	p value	direction
Su1	[38,202]	151.2 ± 47.6	157.2 ± 59.9	3744	0.812	low<high
Su2	[38,111]	210.5 ± 52.2	176.6 ± 59.3	2800	0.003	low>high
Sp1	[624]	139.9 ± 46.0	92.3 ± 43.8	110	0.050	low>high
Sp2	[750]	138.3 ± 38.1	147.9 ± 93.6	176	0.991	low<high

**Fig 12:**
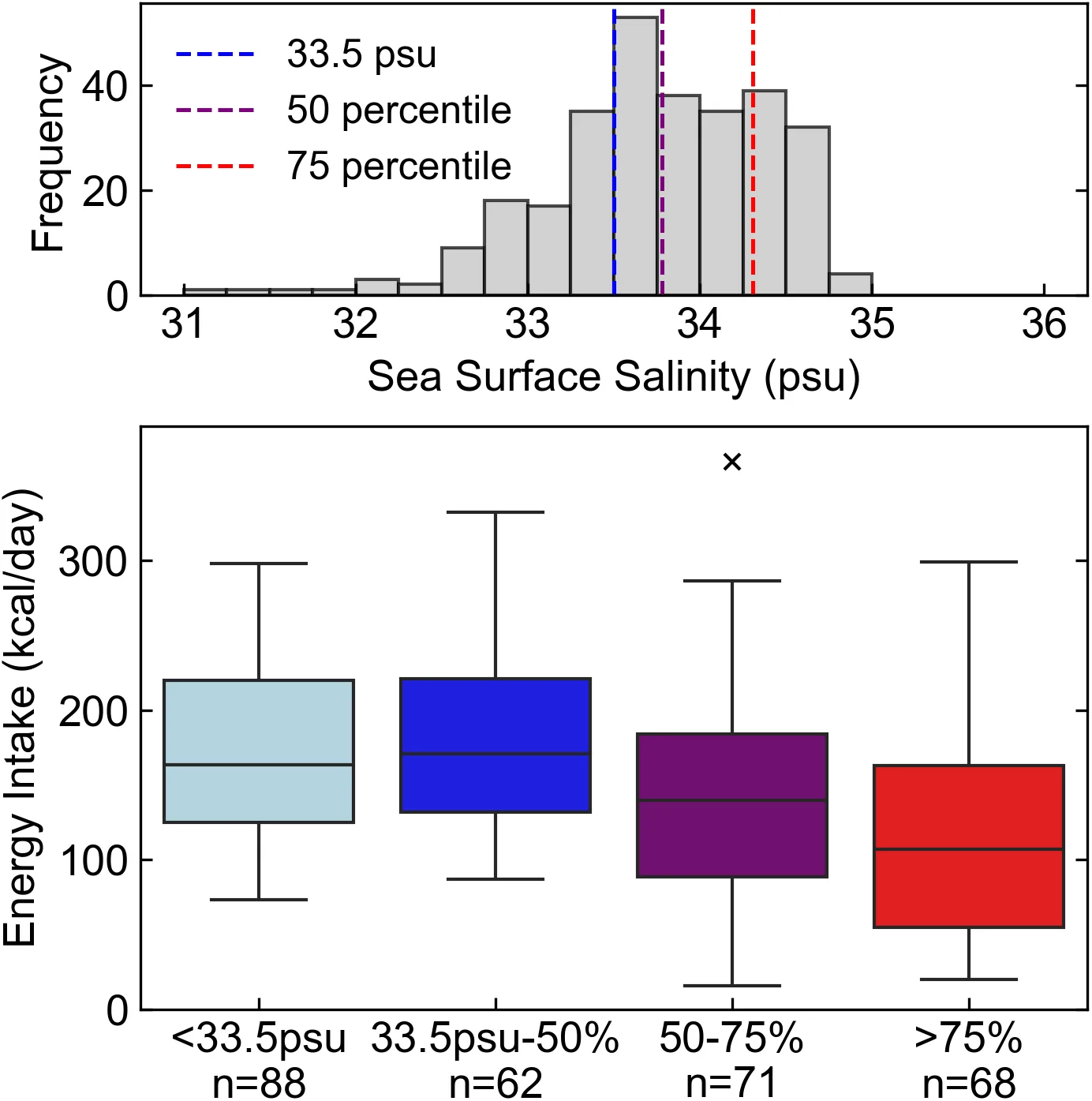
Distribution of daily energy intake of skipjack tuna in relation to SSS. Histograms of SSS is shown in the upper panels. Energy intake values are presented as box plots and categorized into four groups based on SSS (<33.5 psu, ≧ 33.5 psu to 50th percentile, 50th to 75th percentile, and >75th percentile).

## Discussion

The 5th–95th percentile ranges of environmental conditions experienced by all 18 individuals were generally consistent with those reported in previous studies conducted in other oceans (e.g., Northwestern Pacific [9], Southwestern Atlantic [16], Western Central Pacific [12], South Java Sea West [19], and off the coast of Sumatra [18]). However, the lowest SSS recorded in this study was 31.3 psu, which was notably lower than those reported previously (34.9–35.8 psu [16]; 34.29–35.28 psu [12]; 33.075–34.514 psu [19]; and 32.9–34.5 psu [18]). The ECS was characterized by the coexistence of low-salinity environments resulting from freshwater discharge from the Yangtze River [27] and high-salinity environments influenced by the Kuroshio Current [54]. In this study, skipjack tuna migrated primarily within high-salinity environments, consistent with observations in other oceanic regions [12,16,18,19], with only a limited number of individuals utilizing low-salinity environments.

Likewise, SSC data revealed that some individuals experienced high-SSC environments and that a subset of individuals that migrated through low-salinity environments experienced high-primary-productivity environments associated with nutrients supplied by the Yangtze River [33]. Skipjack tuna experienced warm waters where SST was primarily between 22.7°C and 30.5°C. The waters around the Nansei Islands and the ECS were classified as a subtropical ocean. The wider temperature range observed in this study compared with those in other regions (20.5°C–26.0°C [9]; 22.0°C–24.5°C [16]; > 29.6°C [12]; 26.050°C–30.816°C [19]; and 28.5°C–32.2°C [18]) was likely due to significant seasonal variation and the influence of the Kuroshio Current [28,29] and appeared broader than those reported in prior studies.

### Horizontal movement of skipjack tuna in the Nansei Islands and the ECS

Based on the migration patterns of the five individuals that entered the ECS, temperature appears to be a key limiting factor influencing skipjack migration routes in spring. Spring SST (Figs 7g and 7h) dropped to 18°C, corresponding to the reported lower temperature threshold for skipjack [10,26]. In contrast, the ECS during summer was homogeneously warm (>26°C; Figs 7e and 7f). Furthermore, skipjack tuna migration and transit into low-temperature regions across frontal zones reportedly occur within temperature ranges that enable the maintenance of metabolic function [55,56]. Therefore, the observed differences in migration patterns between the spring and summer groups corresponded to seasonal differences in water temperature, although the sample number was limited. In spring, strong ∇SST and the presence of low-temperature waters below 18°C have been associated with the shorter duration of stay in low-salinity waters (approximately one week for the spring group versus over two months for the summer group). Whether this difference reflects active avoidance of low temperatures or other behavioral responses remains to be tested in future studies with plenty of skipjack tuna samples.

Meanwhile, in summer, skipjack could enter the ECS because of the absence of temperature-related limitations. During this period, the lowest recorded SSS was 31.3 psu—the minimum observed in this study. Notably, skipjack did not venture further into the ECS beyond this point and subsequently returned toward the area around the Nansei Islands. Although these results are based on only one individual, this behavior suggests that a lower salinity threshold might exist for skipjack, and salinities below 31.3 psu can directly and indirectly restrict further migration.

Focusing solely on salinity, even lower values have been reported in some regions. Off the west coast of Africa in the Atlantic Ocean, a minimum value of 30.3 psu was reported within skipjack habitat [17]. In this region, although the Congo River supplies freshwater, the study did not discuss riverine impact but rather focused on the effects of upwelling. In coastal waters off Brazil, which also receive major riverine input, the reported salinity range (34.8–35.7 psu [16]) was higher than that observed in this study. Although these studies used fishery data that revealed distributional patterns, the archival tag data captured continuous behavioral changes in skipjack. The difference in minimum salinity values between the ECS and coastal Brazil, despite both regions being influenced by river discharge, may be because the archival tags enabled the tracking of individual skipjack movements and in situ environmental conditions in waters closer to river mouths. However, only a single individual was observed to reach the central ECS in this study. Further investigations are required to obtain physiological insights into skipjack tuna’s responses to salinity fluctuations and to define salinity tolerance thresholds for their habitat suitability.

### Vertical movement differences of skipjack tuna in the ECS

Differences in skipjack swimming depth were observed between the low-salinity waters and the open ocean in spring and summer. In spring group, skipjack swam at shallower depths in low-salinity waters, and the time spent below 50 m drastically decreased. During this period, DO remained above ~4.5 mL L^−1^ down to about 100 m, exceeding the required DO level (3–3.5 mL L^−1^ [26]). In contrast, the temperatures in the upper layer of low-salinity waters were lower, decreasing to less than 18°C at 60 m. This low salinity – lower temperature relationship was supported by the individual-levels analysis (Fig 10). Skipjack tuna and other tuna, such as yellowfin tuna (*Thunnus albacares*) and bigeye tuna (*Thunnus obesus*), possess counter-current heat exchange systems that transfer heat from muscles throughout their bodies via blood circulation, enabling them to maintain body temperature when diving into colder environments [53,56,57]. Moreover, the primary swimming depth of skipjack reportedly shifts along the 18°C isotherm during their northward migration in waters around Japan [10]. Therefore, these observations suggest that the shallower swimming depths recorded in low-salinity waters during spring may be restricted to the vertical temperature gradients and the shoaling of cold water. This aligns with the observation that no individuals entered waters below 18°C in the low-salinity areas of the ECS.

The swimming depth was also shallower in low-salinity waters for the summer group. Although the vertical temperature profiles (ambient temperature) were broadly similar between the ECS and the open ocean side, the ambient temperature in the ECS was generally lower overall, whereas DO decreased sharply below 40 m. The 95th-percentile DO at the depth corresponding to the 95th-percentile swimming depth of skipjack in low-salinity waters for the summer group (66.8 m) was 3.6 mL L^−1^, a value close to the required DO. The DO difference between the low-salinity waters and the open ocean may be attributed to density stratification caused by the Yangtze River discharge, increased phytoplankton production resulting from terrestrial-derived nutrients, and enhanced oxygen consumption by bacteria in the bottom layer [30,31,33].

Previous studies indicate that skipjack swimming depth is constrained by low-DO environments. Skipjack swimming is typically confined to the mixing layer above the thermocline, as the DO levels below the thermocline may be insufficient [58]. Previously, studies found that skipjack tuna spent very little time at depths exceeding 150 m (DO < 3.5 mL L^−1^) around the Comoros Islands [59] and usually remained at depths less than 100 m (DO 2.7–3.2 mL L^−1^) in the equatorial central Pacific [11], suggesting avoidance of deeper, lower-oxygen waters. Meanwhile, diving below the thermocline during the daytime has also been documented [15]. In the present study, skipjack were predominantly observed in upper layers where DO concentrations were relatively high, and significant positive correlations were observed between swimming depth and DO in both summer individuals. Therefore, the shallower swimming depth recorded in low-salinity waters during summer might have been constrained by reduced DO in deeper layers, consistent with the absence of individuals in waters where DO fell below approximately 3.5 mL L ^−^ ¹. Similar depth restrictions in response to low DO have also been reported for yellowfin tuna in the eastern Pacific Ocean [60]. However, whether low DO levels directly affect the physiological state of skipjack or whether changes in prey fish distribution play a role remains unclear. Moreover, these findings may have been either overestimated or underestimated, as the WOA data used in this study represented monthly averages with a 1° spatial resolution and did not account for short-term changes, such as those caused by heavy rain or flooding. More detailed insights are expected through the continued development of archival tags and ongoing ecological and physiological studies of skipjack tuna, and further investigation of prey fish distribution and behavior.

### Energy intake in the ECS

In the low-salinity waters, the estimated energy intake of 2 individuals (Su2, Sp1) tended to increase. The ECS exhibited high primary production in the upper layer due to the nutrient input from the Yangtze River [33,61]. Skipjack migrating to Japan feed along their northward route, and energy intake increases in frontal areas formed by the Kuroshio and Oyashio, where nutrient enrichment creates productive feeding grounds [39,62]. Furthermore, as the feeding environments for skipjack tuna are formed in coastal waters influenced by rivers, such as those along the Brazilian coast [16], similar feeding environments may have also formed in the ECS. Additionally, increased yellowfin tuna catch rates near salinity fronts have been reported in the Western Banda Sea [63]. Therefore, the ECS, being close to the Kuroshio Current and containing low-salinity, nutrient-rich zones, may serve as a favorable feeding environment. In addition, skipjack prey species, such as Japanese jack mackerel (*Trachurus japonicus*) and Japanese anchovy (*Engraulis japonicus*), are widely distributed in the ECS [64,65]. Thus, the ECS is expected to provide significant feeding opportunities for skipjack.

## Conclusion

In this study, we investigated the relationship between skipjack tuna migrating around the Nansei Islands and the ECS and their behavior using data from 18 individuals tagged in field experiments. Various oceanographic data and a model estimating energy intake were used to assess skipjack behavior from both physical and physiological perspectives. The ocean environment data extracted from skipjack positions around the Nansei Islands were similar to findings from previous studies in other oceans, although the lowest SSS observed was relatively lower, reflecting oceanographic features in the ECS influenced by the Yangtze River.

Among the tagged individuals, only 5 out of 18 individuals were exposed to the low-salinity environment in the ECS: two in summer and three in spring. The following results are therefore descriptive and exploratory in nature, and should be interpreted with caution given this limited sample size. In the spring group, low water temperatures appear to have constrained both horizontal migration and swimming depth. In the summer group, although temperatures were warm, the markedly low salinity in the upper layer and reduced DO in the lower layer – attributable to Yangtze River nutrient-rich freshwater input – may have constrained the vertical habitat of skipjack tuna. These seasonal differences in ocean structure corresponded to distinct behavioral patterns in skipjack tuna. Additionally, in some individuals, energy intake tended to be higher in low-salinity conditions, suggesting that environmental variability induced by river water discharge may have enhanced feeding opportunities. It remains unclear whether temperature, salinity, and DO in the ECS directly affects physiological processes in skipjack tuna. Further studies with larger sample sizes are needed to statistically validate the relationships suggested by these observations. In addition, to understand the behavioral and population dynamics of skipjack tuna and other apex predators in response to environmental variability, further elucidation of their physiological and ecological response mechanisms to the ECS environment is essential.

## Supporting information

S1 Fig *P*-values of sea surface parameters (sea surface salinity [SSS] and sea surface temperature [SST]) and archival tag measurements (ambient temperature only) compared with Argo float data (salinity and temperature) and plotted against distance. For the Argo surface data, the shallowest data point from each cycle was used. Dotted lines indicate *P*-value = 0.05.
(TIF)

S2 Fig Shortest distance between skipjack tuna and Argo float measurements recorded within 294 km on the same day. Red and blue dots indicate the estimated positions of skipjack tuna and Argo floats, respectively.
(TIF)

S3 Fig Cumulative number of individuals exposed to the lowest SSS experienced by each skipjack. Red dotted lines indicate 33.5 psu.
(TIF)
